# Supplementary figures and images for: Theta-Phase Connectivity between Medial Prefrontal and Posterior Areas Underlies Novel Instructions Implementation
Source: eNeuro. 2022 Aug 5;9(4):ENEURO.0225-22.2022. doi: 10.1523/ENEURO.0225-22.2022 (PMC9374157; doi:10.1523/ENEURO.0225-22.2022)

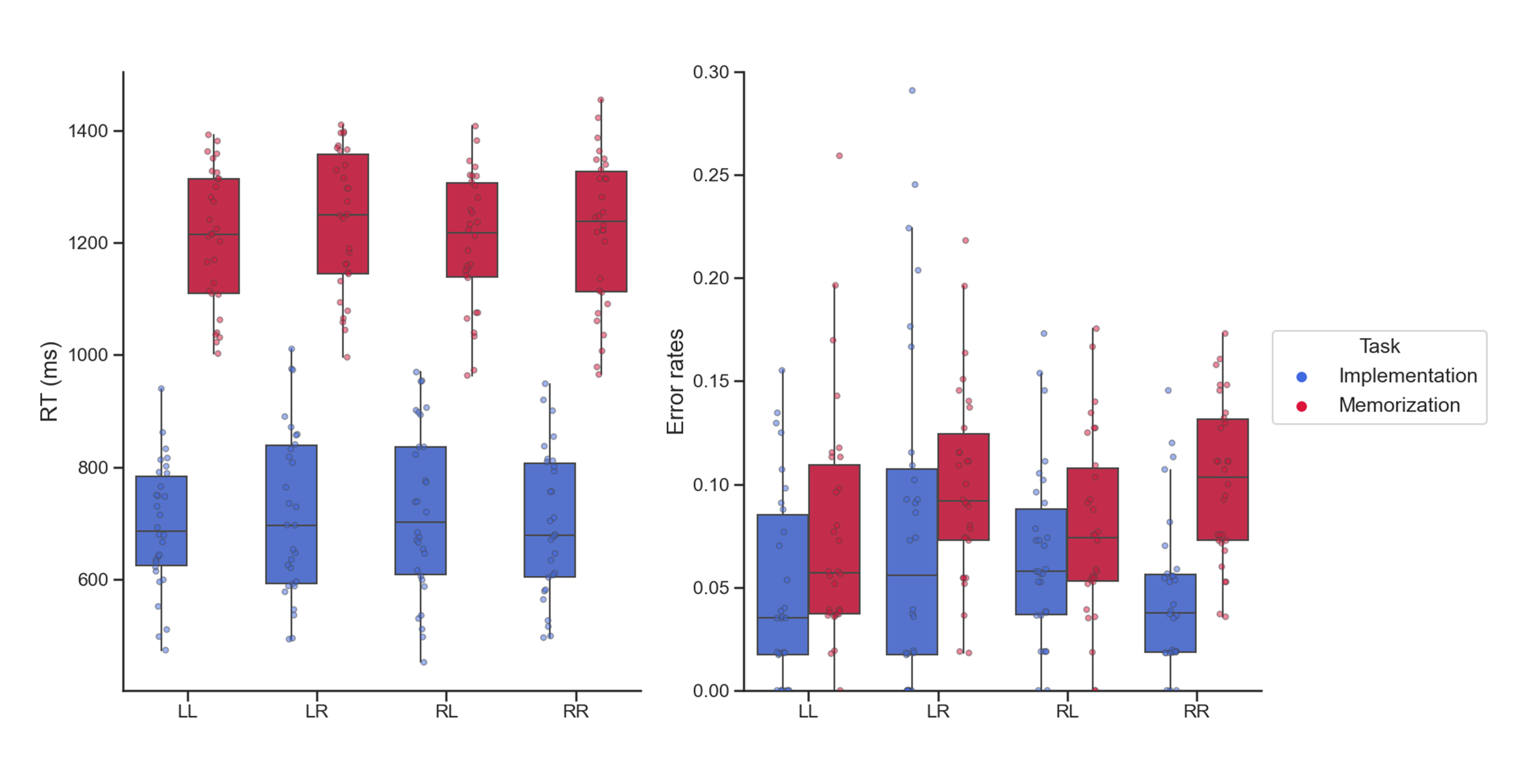

Supplement: Extended Data Figure 3-1 — Exploratory three-way ANOVAs on RTs (left panel) and Error rates (right panel). The exploratory three-way ANOVA on RTs confirmed the main effect of Task (F29,1 = 841.59, p < 0.001, η2p = 0.98) and additionally yielded significant effects of Response Side (F29,1 = 4.74, p = 0.038, η2p = 0.14) and the interaction of Cued * Response Side (F29,1 = 6.19, p = 0.019, η2p = 0.18). The corresponding ANOVA on Error Rates showed significant effects for Task (F29,1 = 13.36, p < 0.001, η2p = 0.31), Response Side (F29,1 = 5.64, p = 0.024, η2p = 0.16), the interaction of Cued * Response Side (F29,1 = 6.50, p = 0.016, η2p = 0.18), and the three-way interaction of Task * Cued Side * Response Side (F29,1 = 7.66, p = 0.010, η2p = 0.21). X-axis labels refer to the individual conditions resulting from the crossing of Cued Side and Response Side. The first letter indicates the cued hemispace (L for Left and R for right), the second letter denotes the instructed response hand (L for Left and R for Right). In each boxplot, the thick line inside box plots depicts the second quartile (median) of the distribution (n = 30). The bounds of the boxes depict the first and third quartiles of the distribution. Whiskers denote the 1.5 interquartile range of the lower and upper quartile. Dots represent individual subjects’ scores. Download Figure 3-1, TIF file. [file enu-eN-NWR-0225-22-s01.tif]

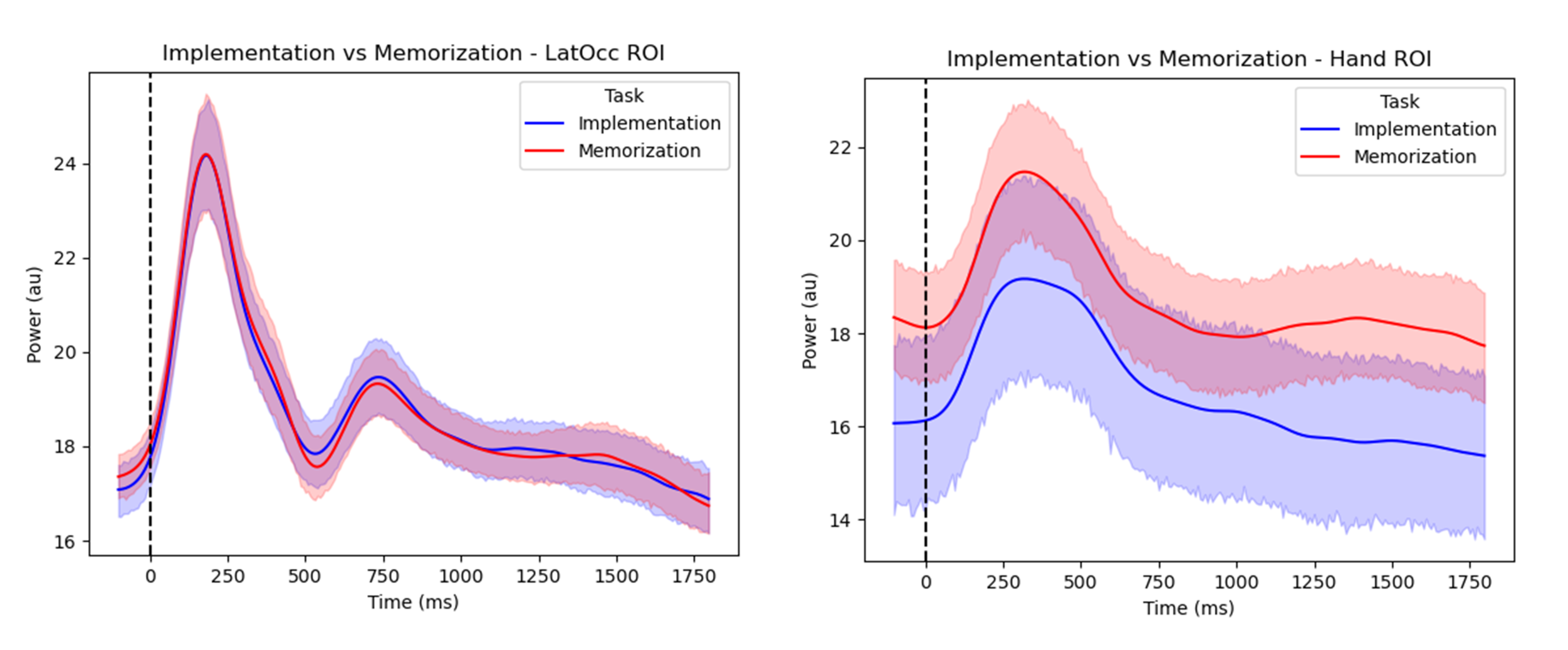

Supplement: Extended Data Figure 6-1 — Theta power in LatOcc (left panel) and Hand (right panel) ROIs. To investigate to what extent our results might be affected by volume conduction, we compared theta power across tasks in the LatOcc and Hand ROIs. The aim of this control analysis was to check whether the task-specific increase in theta oscillations found in mPFC could be detected also from our other ROIs, namely Hand and LatOcc ROIs. To equate this analysis to the original one reported for mPFC, we used bilateral ROIs and compared theta power across tasks by means of a cluster-based permutation. No cluster was observed, supporting our claim that differences in connectivity between mPFC and posterior areas cannot be solely attributed to volume conduction from the former to the latter. The figure shows time courses of theta power, separately for each Task, time-locked to the onset of the retro-cue. Shading indicates the standard error of the mean (s.e.m). Download Figure 6-1, TIF file. [file enu-eN-NWR-0225-22-s02.tif]

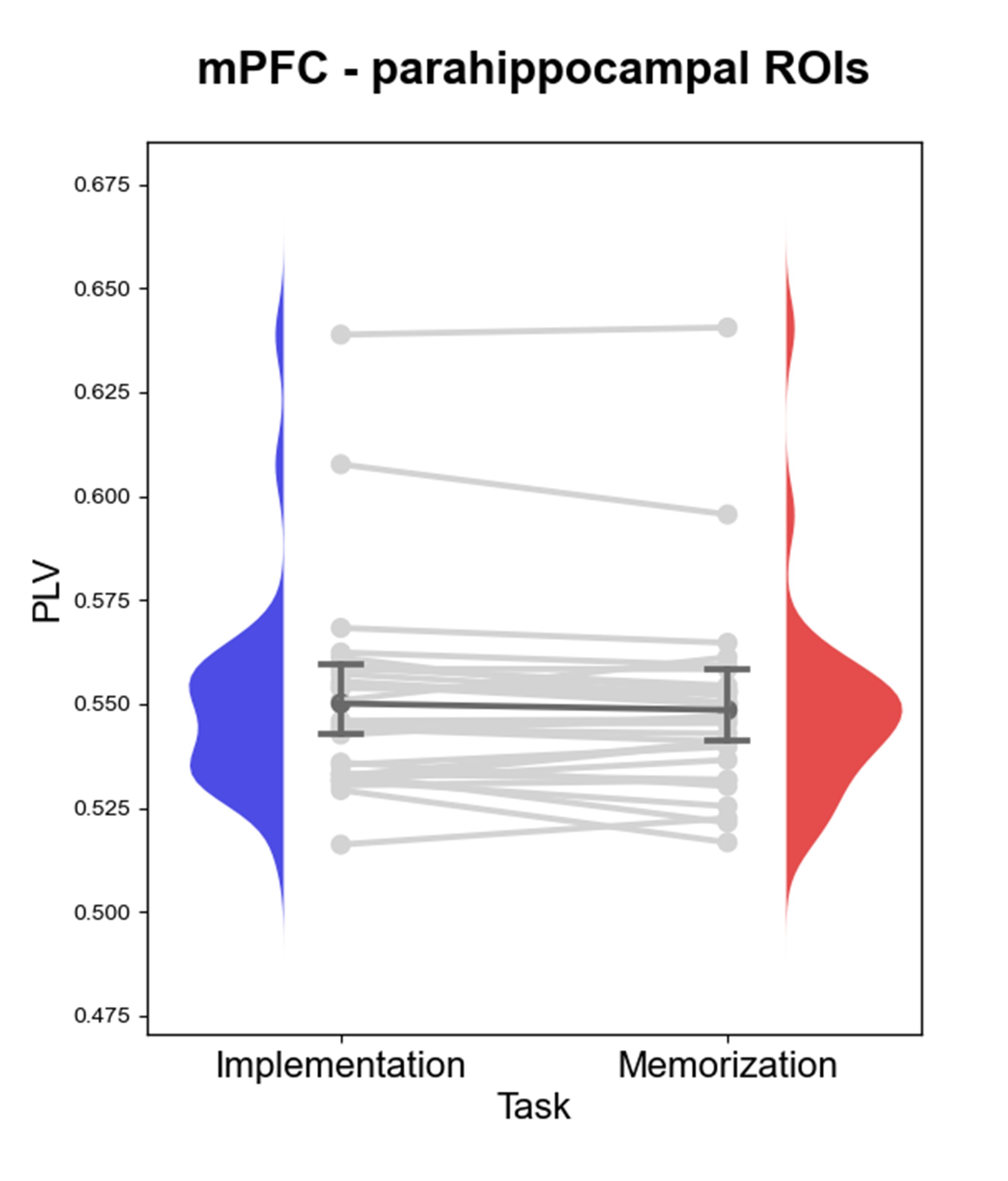

Supplement: Extended Data Figure 8-1 — Connectivity between mPFC and Parahippocampal ROIs. We investigated the connectivity patterns between mPFC and control areas, namely bilateral Parahippocampal ROIs. No task-related differences in synchronization between these regions were observed (F = 1.99, p = 0.17). This control analysis supports the assumption that mPFC selectively synchronizes the activity of task relevant areas, rather than producing a general effect across the whole brain. For visualization purposes, the figure depicts subject-level averages. Blue and red curves represent the density distributions of subject-level averages of PLV of Implementation and Memorization, respectively. Light gray lines connect the average in the two Tasks for each individual participant, whereas the dark gray line connect the group-level averages (whiskers denote 95% confidence intervals). Download Figure 8-1, TIF file. [file enu-eN-NWR-0225-22-s03.tif]

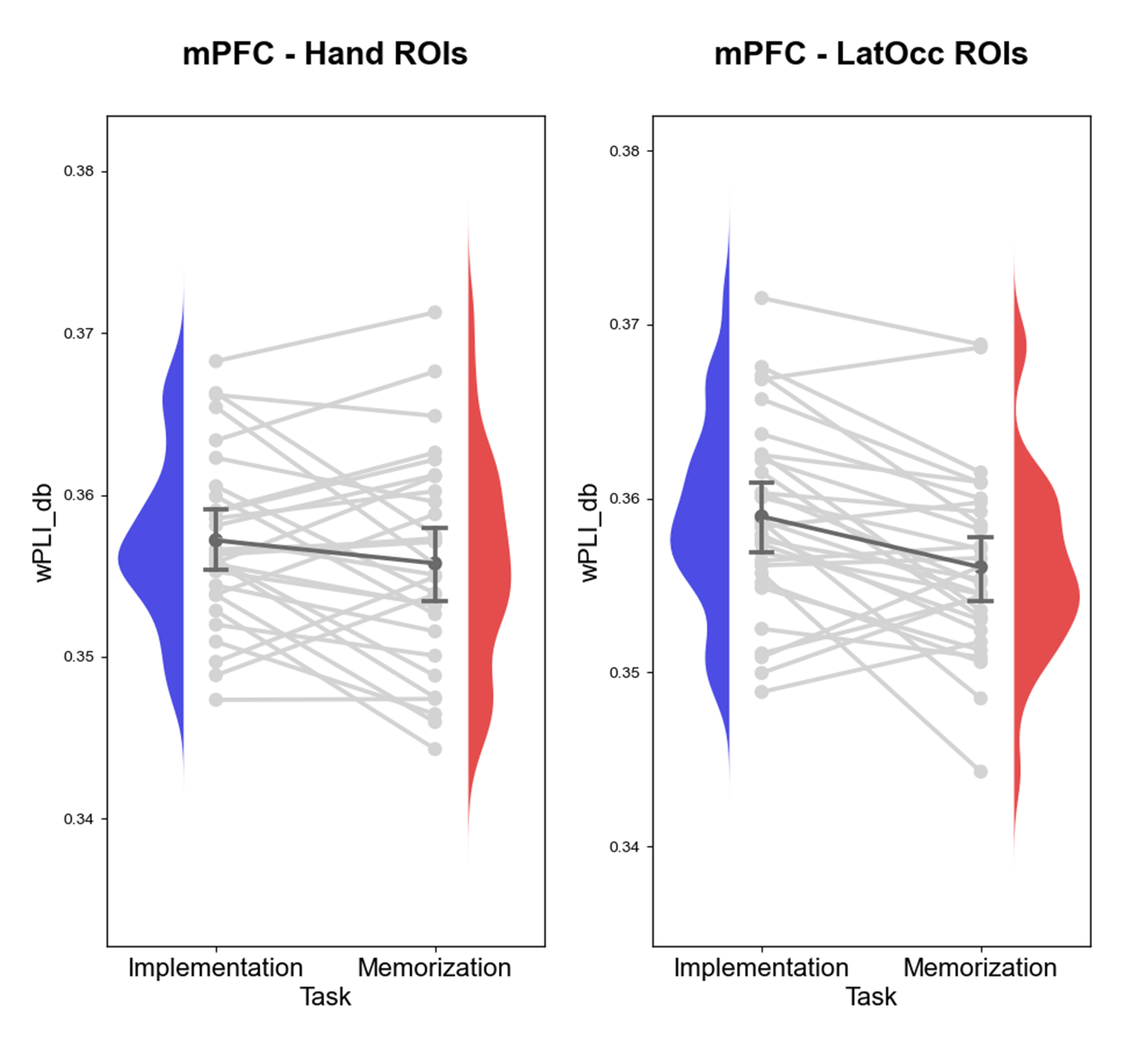

Supplement: Extended Data Figure 8-2 — Connectivity between mPFC and posterior ROIs: wPLI. Connectivity analyses were additionally repeated using the weighted Phase-Lag Index (wPLI), a measure insensitive to volume conduction and source leakage issues, although more prone to false negatives in case of short delays between truly synchronized areas. wPLI values between mPFC and LatOcc ROIs are significantly larger during Implementation.The effect of task-demands in the synchronization between mPFC and Hand ROIs is significant with PLV, and only shows a trend towards significance for the wPLI. Given the higher spatial proximity of mPFC and Hand ROIs (as compared to mPFC and LatOcc ROIs), it is possible that the PLV between their signals is partially affected by volume conduction. At the same time, it is reasonable to speculate that true-connectivity would likely occur at a close-to-zero lag phase consistency, thus leading to the wPLI underestimating the existing phase synchronization However, the overall pattern of results with wPLI is consistent with the PLV, albeit less strong for the pair mPFC-Hand ROIs, and is in line with our hypothesis of stronger synchronization for Implementation task demands. The figure displays wPLI values between mPFC and motor regions (left panel) and visual regions (right panel); the plots depict subject-level averages. Blue and red curves represent the density distributions of subject-level averages of wPLI of Implementation and Memorization, respectively. Light gray lines connect the average in the two Tasks for each individual participant, whereas the dark gray line connect the group-level averages (whiskers denote 95% confidence intervals). Download Figure 8-2, TIF file. [file enu-eN-NWR-0225-22-s04.tif]
